# 2-Amino-4-methyl­pyridinium trifluoro­acetate: a monoclinic polymorph

**DOI:** 10.1107/S1600536810008408

**Published:** 2010-03-17

**Authors:** Mehrdad Pourayoubi, Maryam Toghraee, Arnold L. Rheingold, James A. Golen

**Affiliations:** a Department of Chemistry, Ferdowsi University of Mashhad, Mashhad 91779, Iran; b Department of Chemistry, University of California, San Diego, 9500 Gilman, Drive, La Jolla, CA 92093, USA

## Abstract

The title salt, C_6_H_9_N_2_^+^·C_2_F_3_O_2_^−^, is a monoclinic polymorph of a previously reported structure [Hemamalini & Fun (2010). *Acta Cryst.* E**66**, o781–o782]. In the crystal structure, the cations and anions are linked by two different types of N—H⋯O hydrogen bonds, forming cation–anion pairs. These pairs are hydrogen bonded to neighbouring pairs *via* another N—H⋯O hydrogen bonds involving an H atom of the NH_2_ group and one of the O atoms of the COO^−^ group into a chain extended along the *b* axis.

## Related literature

For a related structure and the triclinic polymorph of the title salt, see: Hemamalini & Fun (2010*a* ▶,*b* ▶).
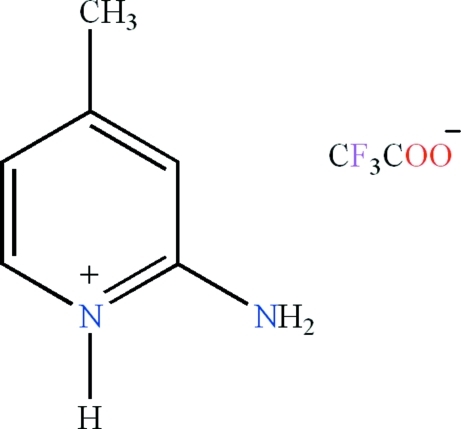


## Experimental

### 

#### Crystal data

C_6_H_9_N_2_^+^·C_2_F_3_O_2_^−^
*M* *_r_* = 222.17
Monoclinic, 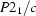

*a* = 8.5315 (7) Å
*b* = 11.4901 (9) Å
*c* = 9.7206 (8) Å
β = 90.820 (1)°
*V* = 952.79 (13) Å^3^
*Z* = 4
Mo *K*α radiation
μ = 0.15 mm^−1^
*T* = 100 K
0.30 × 0.20 × 0.10 mm

#### Data collection

Bruker SMART APEX diffractometer
10752 measured reflections
2197 independent reflections
1784 reflections with *I* > 2σ(*I*)
*R*_int_ = 0.036

#### Refinement

*R*[*F* ^2^ > 2σ(*F* ^2^)] = 0.035
*wR*(*F* ^2^) = 0.098
*S* = 1.06
2197 reflections
150 parameters
H atoms treated by a mixture of independent and constrained refinement
Δρ_max_ = 0.29 e Å^−3^
Δρ_min_ = −0.23 e Å^−3^

### 

Data collection: *SMART* (Bruker, 2005 ▶); cell refinement: *SAINT* (Bruker 2005 ▶); data reduction: *SAINT*; program(s) used to solve structure: *SHELXS97* (Sheldrick, 2008 ▶); program(s) used to refine structure: *SHELXL97* (Sheldrick, 2008 ▶); molecular graphics: *SHELXTL* (Sheldrick, 2008 ▶); software used to prepare material for publication: *SHELXTL*.

## Supplementary Material

Crystal structure: contains datablocks I, global. DOI: 10.1107/S1600536810008408/ng2740sup1.cif

Structure factors: contains datablocks I. DOI: 10.1107/S1600536810008408/ng2740Isup2.hkl

Additional supplementary materials:  crystallographic information; 3D view; checkCIF report

## Figures and Tables

**Table 1:** Hydrogen-bond geometry (Å, °)

*D*—H⋯*A*	*D*—H	H⋯*A*	*D*⋯*A*	*D*—H⋯*A*
N1—H1*B*⋯O2	0.952 (19)	1.82 (2)	2.7724 (14)	177.4 (18)
N2—H2*C*⋯O1	0.901 (18)	1.938 (19)	2.8376 (15)	176.3 (17)
N2—H2*B*⋯O2^i^	0.923 (18)	2.055 (18)	2.8946 (15)	150.5 (15)

Symmetry code: (i) 
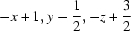
.

**Fig. 1.**
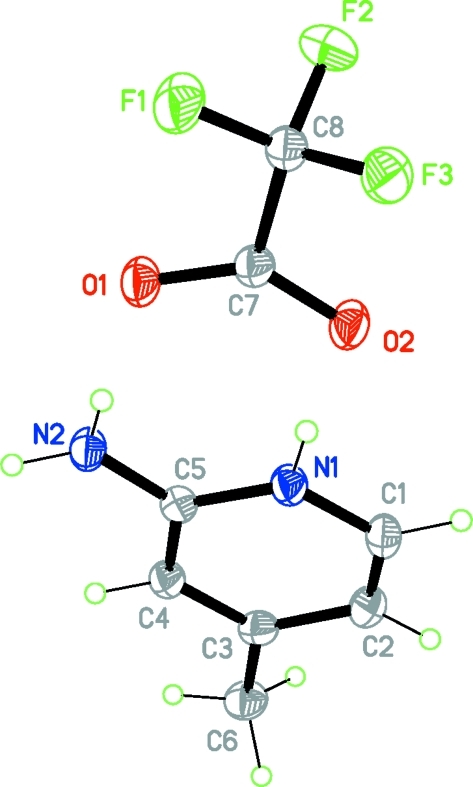
The molecular structure of the title salt, showing the atom-numbering scheme. Displacement ellipsoids are drawn at the 50 % probability level.

**Fig. 2.**
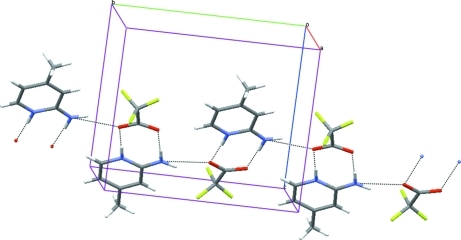
A view of crystal packing along the b axis, hydrogen bonds are shown by dashed lines.

## supplementary crystallographic information

### Comment

In the previous works, the structure determinations of 2-aminopyridinium trifluoroacetate and the triclinic polymorph of 2-amino-4-methylpyridinium trifluoroacetate (Hemamalini & Fun, 2010*a*,*b*) have been investigated; we report here on the crystal structure of title compound, 4-methyl-2-aminopyridinium trifluoroacetate (Fig. 1). The cation and anion are linked by two different types of N—H···O hydrogen bonds, forming the cation-anion pair. The pairs are hydrogen bonded to neighbouring pairs *via* another N—H···O hydrogen bonds between the hydrogen of NH_2_ moiety and one of the oxygen atom of COO^-^ group into chain extended along the *b* axis (Fig. 2).

### Experimental

The title compound was obtained accidently from the reaction between 2,2,2-trifluoroacetamide, phosphorus pentachloride and formic acid and then the treatment of 2-amino-4-methylpyridine and triethylamine. The crystal was obtained from chloroform and n-heptane at room temperature.

### Refinement

The H atoms of the NH2 group were located from the difference Fourier synthesis and refined isotropically, no restraints were used. Finally, the geometrical and thermal parameters obtained for these H-atoms, as well as parameters of the hydrogen bonds for these H-atoms included, were rather realistic. The H(C) atom positions were calculated and refined in isotropic approximation in riding model with the Uiso(H) parameters equal to 1.2 Ueq(Ci) for the aromatic C atoms, for methyl groups equal to 1.5 Ueq(Cii), where U(Ci) and U(Cii) are respectively the equivalent thermal parameters of the carbon atoms to which corresponding H atoms are bonded.

### Crystal data

**Table:**

C_6_H_9_N_2_^+^·C_2_F_3_O_2_^−^	*F*(000) = 456
*M**_r_* = 222.17	*D*_x_ = 1.549 Mg m^−^^3^
Monoclinic, *P*2_1_/*c*	Mo *K*α radiation, λ = 0.71073 Å
Hall symbol: -P 2ybc	Cell parameters from 5265 reflections
*a* = 8.5315 (7) Å	θ = 2.7–28.0°
*b* = 11.4901 (9) Å	µ = 0.15 mm^−^^1^
*c* = 9.7206 (8) Å	*T* = 100 K
β = 90.820 (1)°	Block, colorless
*V* = 952.79 (13) Å^3^	0.30 × 0.20 × 0.10 mm
*Z* = 4	

### Data collection

**Table:**

Bruker SMART APEX diffractometer	1784 reflections with *I* > 2σ(*I*)
Radiation source: fine-focus sealed tube	*R*_int_ = 0.036
graphite	θ_max_ = 28.2°, θ_min_ = 2.4°
φ and ω scans	*h* = −5→11
10752 measured reflections	*k* = −14→14
2197 independent reflections	*l* = −12→12

### Refinement

**Table:**

Refinement on *F*^2^	Secondary atom site location: difference Fourier map
Least-squares matrix: full	Hydrogen site location: inferred from neighbouring sites
*R*[*F*^2^ > 2σ(*F*^2^)] = 0.035	H atoms treated by a mixture of independent and constrained refinement
*wR*(*F*^2^) = 0.098	*w* = 1/[σ^2^(*F*_o_^2^) + (0.0474*P*)^2^ + 0.2454*P*] where *P* = (*F*_o_^2^ + 2*F*_c_^2^)/3
*S* = 1.06	(Δ/σ)_max_ = 0.001
2197 reflections	Δρ_max_ = 0.29 e Å^−^^3^
150 parameters	Δρ_min_ = −0.23 e Å^−^^3^
0 restraints	Extinction correction: *SHELXL97* (Sheldrick, 2008), Fc^*^=kFc[1+0.001xFc^2^λ^3^/sin(2θ)]^-1/4^
Primary atom site location: structure-invariant direct methods	Extinction coefficient: 0.0084 (16)

### Special details

**Table:**

Geometry. All esds (except the esd in the dihedral angle between two l.s. planes) are estimated using the full covariance matrix. The cell esds are taken into account individually in the estimation of esds in distances, angles and torsion angles; correlations between esds in cell parameters are only used when they are defined by crystal symmetry. An approximate (isotropic) treatment of cell esds is used for estimating esds involving l.s. planes.
Refinement. Refinement of *F*^2^ against ALL reflections. The weighted *R*-factor wR and goodness of fit *S* are based on *F*^2^, conventional *R*-factors *R* are based on *F*, with *F* set to zero for negative *F*^2^. The threshold expression of *F*^2^ > σ(*F*^2^) is used only for calculating *R*-factors(gt) etc. and is not relevant to the choice of reflections for refinement. *R*-factors based on *F*^2^ are statistically about twice as large as those based on *F*, and *R*- factors based on ALL data will be even larger.

### Fractional atomic coordinates and isotropic or equivalent isotropic displacement parameters (Å^2^)

**Table:**

	*x*	*y*	*z*	*U*_iso_*/*U*_eq_	
F1	0.16929 (11)	0.27679 (7)	1.06142 (10)	0.0350 (2)	
F2	0.08357 (10)	0.40806 (8)	0.92121 (10)	0.0358 (2)	
F3	0.25375 (10)	0.45302 (7)	1.07851 (9)	0.0316 (2)	
O1	0.36035 (11)	0.23798 (8)	0.85540 (11)	0.0259 (2)	
O2	0.41240 (11)	0.42938 (8)	0.83832 (10)	0.0257 (2)	
N1	0.61429 (13)	0.37742 (9)	0.62595 (12)	0.0212 (2)	
H1B	0.544 (2)	0.3930 (17)	0.699 (2)	0.048 (5)*	
N2	0.56684 (13)	0.18015 (10)	0.63958 (12)	0.0223 (3)	
H2C	0.504 (2)	0.1964 (16)	0.711 (2)	0.040 (5)*	
H2B	0.588 (2)	0.1040 (16)	0.6158 (18)	0.034 (5)*	
C1	0.68436 (16)	0.47274 (12)	0.56989 (15)	0.0251 (3)	
H1A	0.6618	0.5480	0.6049	0.030*	
C2	0.78596 (16)	0.46100 (12)	0.46475 (15)	0.0250 (3)	
H2A	0.8341	0.5277	0.4261	0.030*	
C3	0.81986 (15)	0.34848 (12)	0.41287 (14)	0.0214 (3)	
C4	0.74763 (14)	0.25417 (11)	0.47012 (13)	0.0206 (3)	
H4A	0.7689	0.1784	0.4359	0.025*	
C5	0.64122 (14)	0.26797 (11)	0.58005 (14)	0.0196 (3)	
C6	0.93335 (16)	0.33574 (13)	0.29694 (15)	0.0260 (3)	
H6A	0.9287	0.2560	0.2612	0.039*	
H6B	1.0398	0.3523	0.3307	0.039*	
H6C	0.9055	0.3905	0.2233	0.039*	
C7	0.34308 (14)	0.34184 (11)	0.88424 (14)	0.0200 (3)	
C8	0.21258 (15)	0.36940 (11)	0.98849 (15)	0.0236 (3)	

### Atomic displacement parameters (Å^2^)

**Table:**

	*U*^11^	*U*^22^	*U*^33^	*U*^12^	*U*^13^	*U*^23^
F1	0.0412 (5)	0.0257 (5)	0.0385 (5)	−0.0036 (4)	0.0209 (4)	0.0027 (4)
F2	0.0220 (4)	0.0367 (5)	0.0488 (6)	0.0070 (3)	0.0017 (4)	−0.0026 (4)
F3	0.0328 (5)	0.0271 (5)	0.0350 (5)	−0.0012 (3)	0.0096 (4)	−0.0114 (4)
O1	0.0294 (5)	0.0168 (5)	0.0319 (6)	−0.0008 (4)	0.0099 (4)	−0.0027 (4)
O2	0.0292 (5)	0.0179 (5)	0.0303 (6)	−0.0033 (4)	0.0105 (4)	0.0005 (4)
N1	0.0223 (5)	0.0177 (5)	0.0237 (6)	0.0006 (4)	0.0044 (4)	−0.0015 (5)
N2	0.0250 (6)	0.0168 (6)	0.0253 (6)	−0.0007 (4)	0.0073 (5)	−0.0007 (5)
C1	0.0272 (7)	0.0168 (6)	0.0314 (8)	−0.0007 (5)	0.0026 (6)	−0.0014 (5)
C2	0.0250 (7)	0.0200 (7)	0.0302 (8)	−0.0033 (5)	0.0032 (5)	0.0040 (5)
C3	0.0181 (6)	0.0239 (7)	0.0223 (7)	−0.0005 (5)	0.0013 (5)	0.0008 (5)
C4	0.0206 (6)	0.0187 (6)	0.0224 (7)	0.0006 (5)	0.0019 (5)	−0.0010 (5)
C5	0.0185 (6)	0.0181 (6)	0.0221 (6)	0.0003 (4)	−0.0005 (5)	−0.0005 (5)
C6	0.0236 (7)	0.0298 (7)	0.0247 (7)	−0.0021 (5)	0.0052 (5)	0.0023 (6)
C7	0.0200 (6)	0.0185 (6)	0.0217 (7)	−0.0005 (5)	0.0022 (5)	−0.0010 (5)
C8	0.0227 (6)	0.0182 (6)	0.0299 (7)	−0.0008 (5)	0.0060 (5)	−0.0015 (5)

### Geometric parameters (Å, °)

**Table:**

F1—C8	1.3337 (15)	C1—H1A	0.9500
F2—C8	1.3475 (16)	C2—C3	1.4190 (19)
F3—C8	1.3429 (16)	C2—H2A	0.9500
O1—C7	1.2352 (15)	C3—C4	1.3688 (18)
O2—C7	1.2523 (15)	C3—C6	1.5033 (18)
N1—C5	1.3552 (16)	C4—C5	1.4209 (17)
N1—C1	1.3650 (17)	C4—H4A	0.9500
N1—H1B	0.952 (19)	C6—H6A	0.9800
N2—C5	1.3290 (16)	C6—H6B	0.9800
N2—H2C	0.901 (18)	C6—H6C	0.9800
N2—H2B	0.923 (18)	C7—C8	1.5490 (18)
C1—C2	1.356 (2)		
			
C5—N1—C1	122.40 (12)	N2—C5—N1	118.49 (12)
C5—N1—H1B	122.1 (12)	N2—C5—C4	123.85 (12)
C1—N1—H1B	115.5 (12)	N1—C5—C4	117.67 (12)
C5—N2—H2C	118.0 (12)	C3—C6—H6A	109.5
C5—N2—H2B	121.1 (11)	C3—C6—H6B	109.5
H2C—N2—H2B	120.5 (16)	H6A—C6—H6B	109.5
C2—C1—N1	120.62 (12)	C3—C6—H6C	109.5
C2—C1—H1A	119.7	H6A—C6—H6C	109.5
N1—C1—H1A	119.7	H6B—C6—H6C	109.5
C1—C2—C3	119.61 (12)	O1—C7—O2	129.54 (12)
C1—C2—H2A	120.2	O1—C7—C8	115.80 (11)
C3—C2—H2A	120.2	O2—C7—C8	114.62 (11)
C4—C3—C2	118.79 (12)	F1—C8—F3	107.24 (12)
C4—C3—C6	121.75 (12)	F1—C8—F2	106.89 (11)
C2—C3—C6	119.46 (12)	F3—C8—F2	106.54 (10)
C3—C4—C5	120.90 (12)	F1—C8—C7	113.06 (11)
C3—C4—H4A	119.5	F3—C8—C7	112.88 (11)
C5—C4—H4A	119.5	F2—C8—C7	109.85 (11)
			
C5—N1—C1—C2	−0.3 (2)	C3—C4—C5—N2	179.68 (13)
N1—C1—C2—C3	−0.1 (2)	C3—C4—C5—N1	−0.10 (19)
C1—C2—C3—C4	0.5 (2)	O1—C7—C8—F1	18.42 (18)
C1—C2—C3—C6	−179.60 (13)	O2—C7—C8—F1	−163.72 (12)
C2—C3—C4—C5	−0.37 (19)	O1—C7—C8—F3	140.38 (12)
C6—C3—C4—C5	179.73 (12)	O2—C7—C8—F3	−41.76 (17)
C1—N1—C5—N2	−179.33 (12)	O1—C7—C8—F2	−100.87 (14)
C1—N1—C5—C4	0.46 (19)	O2—C7—C8—F2	76.99 (14)

### Hydrogen-bond geometry (Å, °)

**Table:**

*D*—H···*A*	*D*—H	H···*A*	*D*···*A*	*D*—H···*A*
N1—H1B···O2	0.952 (19)	1.82 (2)	2.7724 (14)	177.4 (18)
N2—H2C···O1	0.901 (18)	1.938 (19)	2.8376 (15)	176.3 (17)
N2—H2B···O2^i^	0.923 (18)	2.055 (18)	2.8946 (15)	150.5 (15)

Symmetry codes: (i) −*x*+1, *y*−1/2, −*z*+3/2.

## References

[bb1] Bruker (2005). *SMART* and *SAINT* Bruker AXS Inc., Madison, Wisconsin, USA.

[bb2] Hemamalini, M. & Fun, H.-K. (2010*a*). *Acta Cryst.* E**66**, o691–o692. 10.1107/S1600536810006392 PMC2983566 21580433

[bb4] Hemamalini, M. & Fun, H.-K. (2010*b*). *Acta Cryst.* E**66**, o781–o782. 10.1107/S1600536810008202 PMC2984062 21580622

[bb3] Sheldrick, G. M. (2008). *Acta Cryst.* A**64**, 112–122. 10.1107/S0108767307043930 18156677

